# Curcumin-loaded albumin submicron particles with potential as a cancer therapy: an in vitro study

**DOI:** 10.3762/bjnano.14.93

**Published:** 2023-11-21

**Authors:** Nittiya Suwannasom, Netsai Sriaksorn, Chutamas Thepmalee, Krissana Khoothiam, Ausanai Prapan, Hans Bäumler, Chonthida Thephinlap

**Affiliations:** 1 Division of Biochemistry, School of Medical Sciences, University of Phayao 56000, Thailand; 2 Department of Radiological Technology, Faculty of Allied Health Sciences, Naresuan University, Phitsanulok 65000, Thailand; 3 Division of Microbiology, School of Medical Sciences, University of Phayao 56000, Thailand; 4 Institute of Transfusion Medicine, Charité-Universitätsmedizin Berlin, 10117 Berlin, Germany

**Keywords:** albumin submicron particles, cancer therapy, curcumin, drug delivery

## Abstract

Curcumin (CUR), a polyphenolic compound, shows promising biological properties, particularly antioxidant activity. However, its medical applications are limited due to its low water solubility, bioavailability, and pH-instability. CUR-loaded albumin microparticles (CUR-HSA-MPs) of submicron size in the range of 800 to 900 nm and a zeta potential of −15 mV were prepared. The CUR loading efficiency was up to 65%. A maximum release of 37% of the encapsulated CUR was observed within 6 h when the CUR-HSA-MPs were dispersed in 50% ethanol in PBS at pH 7, while in RPMI 1640 medium the release was 7%. This demonstrates a sustainable release. The in vitro cytotoxicity of CUR-HSA-MPs showed promising anticancer potential against human hepatocellular carcinoma (Huh-7) and human breast adenocarcinoma (MCF-7) cell lines, although this effect was less pronounced in human dermal fibroblasts (HDFB) and human cholangiocyte (MMN) cell lines. Confocal microscopy was used to confirm the uptake of CUR-HSA-MPs by cancer cells. Our studies revealed that HSA-MPs are potentially promising vehicles for increasing the solubility and bioavailability of CUR.

## Introduction

Curcumin (CUR) is a hydrophobic yellow-orange polyphenol compound isolated from the rhizomes of *Curcuma longa*. Numerous studies have consistently demonstrated that CUR displays a vast range of pharmacological and medicinal properties, including antioxidant, anti-inflammatory, anti-diabetic, anti-hepatotoxic activities, and especially anticancer potential [1–2]. Several in vivo and in vitro studies in recent years have demonstrated that CUR can influence cancer cell proliferation, invasion, angiogenesis, and metastasis [3]. It has been reported that CUR exerts anticancer effects in human breast cancer cells (MCF-7) by regulating the expression through a miRNA-mediated mechanism [4]. Furthermore, recent studies have shown that CUR induces apoptosis in human hepatocellular carcinoma cells (Huh-7) by activating p38, leading to FasL-associated apoptosis [5]. However, the clinical application of CUR is restricted by pharmacokinetic drawbacks such as poor water solubility resulting in low bioavailability, low absorption, rapid metabolism, and elimination [6–7]. The reported solubility of free CUR in distilled water at 25 °C is 1.34 µg/mL [8].

The conjugation of CUR with proteins has been confirmed to improve its aqueous stability and solubility [9]. The complexation occurs mainly through hydrophobic interactions in protein cavities [10–11]. In a recent study, zein nanoparticles loaded with CUR have been studied for their potential in treating brain tumors, and the results have demonstrated a reduction in the proliferation and migration of C6 glioma cells in vitro [12]. Other nanoparticles that are being explored as CUR formulations for various applications include silk-based nanoparticles. These are an excellent candidate with longer plasma half-life and slower release rate, indicating high bioavailability [13]. The silk core–shell nanoparticles show high cytotoxicity and cellular uptake regarding breast cancer cells [14]. However, the effectiveness of zein nanoparticles as a delivery vehicle is limited by their poor stability, as they tend to aggregate when suspended in water [15]. Lyophilizing the particles, which is a common process in the preparation of nanoparticle formulations, also causes them to agglomerate due to their hydrophobic properties [16]. In the case of silk nanoparticles, the addition of simple functional groups has provided a limited range of options for chemical modification [17]. In addition, the removal of sericin, which requires a degumming process, is a prerequisite for using silk cocoons in biomedical applications to reduce the risk of an inflammatory response [18]. However, this degumming process can lead to damage to the silk structure, increase the polydispersity of the silk solution produced by reverse engineering, and make quality control more challenging [19]. Additionally, the degummed silk tends to aggregate when stored for long periods in an aqueous solution, typically for weeks at room temperature and for months at 4 °C [20].

Amongst various carriers, albumin seems to be a more attractive biomaterial because of its various advantages such as non-toxic, non-immunogenic, biodegradable, and biocompatible properties, as well as long-term storage stability. In addition to these aspects, it is an abundant, renewable, and cheap resource that is easy to scale up for industrial manufacturing and application and has therefore attracted researchers [21]. Moreover, because of its various drug-binding sites, drugs can effectively be incorporated into the hydrophobic or hydrophilic core of albumin particles. It has been demonstrated that human serum albumin (HSA) acts as a carrier of CUR through direct binding [22]. Kar et al. evaluated the affinity of HSA molecules to interact with CUR and reported that the binding constant was about 1.74 × 10^5^ M^−1^, suggesting a strong interaction [23]. Therefore, albumin can be considered a useful carrier in drug delivery systems for poorly water-soluble CUR.

An effective submicron particle fabrication technique, namely co-precipitation–cross-linking–dissolution (CCD), has been established to produce biopolymer particles [24–26]. In this method, biopolymer particles are produced via co-precipitation, by simply mixing MnCl_2_ and Na_2_CO_3_ solutions in the presence of albumin [26–27]. This approach has been shown to have high effectiveness for entrapping various biopolymers (e.g., proteins and hemoglobin) into carbonate particles before dissolving with EDTA to obtain pure biopolymer submicron particles. The CCD technique is a recently developed method of encapsulation to produce high-quality submicron albumin particles loaded with riboflavin [28] or doxorubicin [29].

In this work, our aim was to determine the ability of albumin microparticles to load CUR by adsorption and to investigate binding with and release from the microparticles. The incorporation of CUR within the versatile biomolecular platform MnCO_3_-HSA-MPs has not been previously reported. The obtained albumin microparticles are expected to enhance the water solubility of CUR, provide controlled release, and improve its biological activity. These peanut-shaped particles are easily synthesized via cost-effective co-precipitation methods. These techniques are simple, low-cost, and easily scalable, enabling precise control over particle characteristics, including size and morphology, and use inexpensive materials without generating toxic waste. Having established an effective delivery system, our second aim was to evaluate the cytotoxicity effect of CUR-HSA-MPs in vitro and determine cell uptake in MCF-7 cell line.

## Results and Discussion

### Preparation of CUR-loaded human serum albumin microparticles

**Figure 1:**
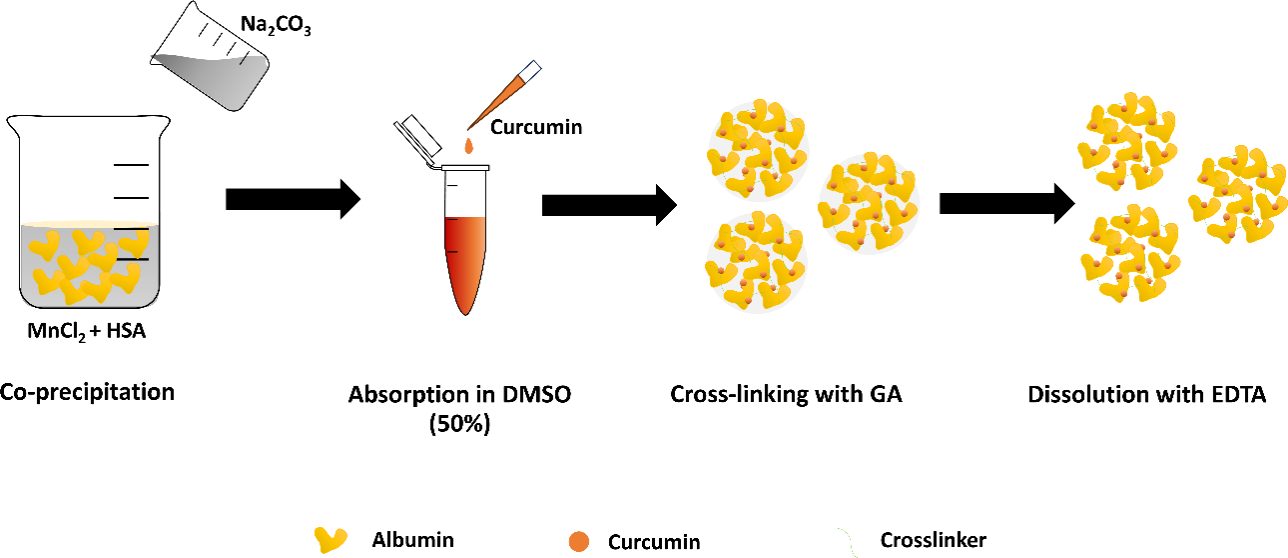
Scheme of the synthesis of the CUR-loaded HSA microparticles via CCD with some modifications.

The preparation process of CUR-loaded human serum albumin microparticles (CUR-HSA-MPs) is shown in Figure 1. At first, MnCO_3_-HSA-MPs were obtained by co-precipitation for the entrapment of HSA. The entrapment efficiency (%EE) of HSA was between 55% and 62% (i.e., the absolute amounts of entrapped HSA in HSA-MPs were up to 31.3 ± 1.8 mg). CUR was then adsorbed onto the MnCO_3_-HSA-MPs and HSA was cross-linked. After dissolution of the MnCO_3_ template with EDTA, a colloidal particle suspension of CUR-HSA-MPs was obtained.

**Figure 2:**
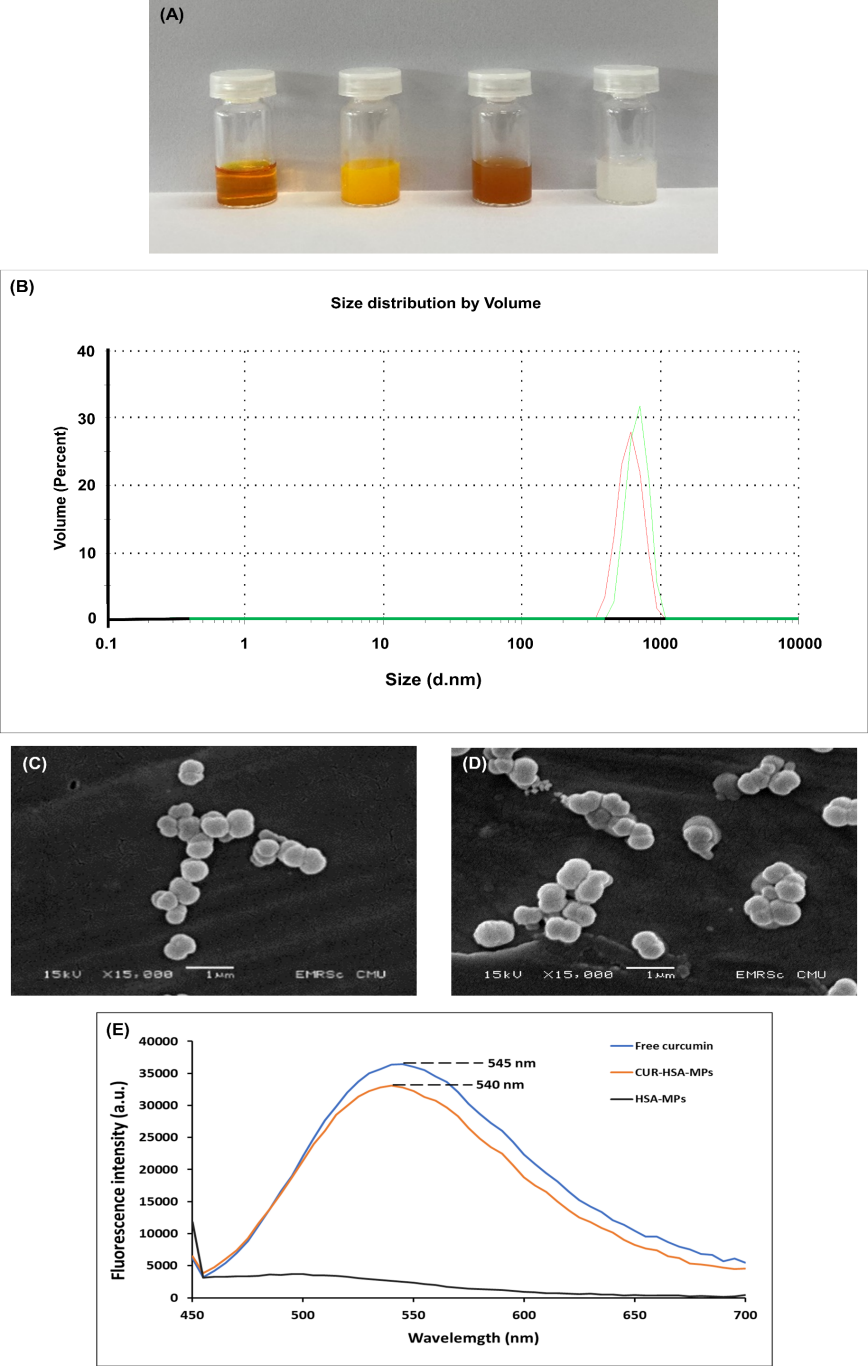
Characterization of curcumin-loaded albumin microparticles. (A) Photographs of sample solutions. From left to right: 1 mL of CUR in DMSO (0.6 mg/mL), CUR in distilled water (0.6 mg/mL), 1 mL of CUR-HSA-MPs loaded with 0.6 mg of CUR in distilled water (0.6 mg/mL), and 1 mL of HSA-MPs (control with an equivalent concentration to CUR-HSA-MPs) in distilled water; (B) the particle size distribution of HSA-MPs (red line) and CUR-HSA-MPs (green line) determined by DLS; SEM images of (C) HSA-MPs and (D) CUR-HSA-MPs; (E) fluorescence spectra of free CUR and CUR-HSA-MPs.

Comparison of the different CUR samples demonstrated that the color of CUR in DMSO is light brown, indicating dissolved CUR; CUR in water is bright yellow and turbid, corresponding to aggregated CUR. CUR incorporated into HSA-MPs yields a colloidal solution of yellowish-brown color, indicating CUR absorption into porous HSA-MPs. In contrast, control albumin particles have a creamy-white color (see below in Figure 2A). Forming a turbid suspension, CUR-HSA-MPs yielded CUR concentrations of 0.55–0.60 mg/mL as estimated by absorbance measurements, which is a more than 400-fold increase in solubility compared to free CUR (1.34 µg/mL [8]).

Due to its hydrophobic nature, CUR aggregates in aqueous solution and interacts with the hydrophobic clusters of albumin proteins [30]. CUR showed strong binding affinity towards HSA, likely at the tryptophan residue, which is present in the hydrophobic cavities of HSA [23,31]. The final CUR-HSA-MPs are obtained after dissolution of the MnCO_3_ template with EDTA.

### Characterization of CUR-HSA-MPs

Dynamic light scattering (DLS) (Figure 2B) shows that the hydrodynamic diameter of HSA-MPs ranged from 800 to 900 nm (888 ± 64 nm) with a PDI value of 0.30. These results are similar to those of CUR-HSA-MPs, which ranged from 900 to 1000 nm (983.57 ± 82 nm) in size with a PDI value of 0.31 (Supporting Information File 1, Table S1). CUR loading did not change significantly the particle size. The particles were quite monodisperse as indicated by the relatively low PDI values.

In addition, the zeta potential values of CUR-HSA-MPs (−15.30 ± 0.02 mV) and HSA-MPs (−14.50 ± 0.06 mV) were similar. The zeta potential analysis of HSA-MPs reflects the negative charge of the albumin biopolymers [26]. In fact, in the cationic HSA-MPs with negative charges, CUR loading did not alter the zeta potential of CUR-HSA-MPs. Furthermore, particles with negative surface charge exhibit the enhanced permeability and retention (EPR) effect together with evasion from recognition by macrophages and the immune system because of the low binding of proteins on the particles’ surface [32].

Representative SEM images of HSA-MPs and CUR-HSA-MPs are displayed in Figure 2C,D. The results reveal that the submicron particles displayed a “hairy” surface. Also, it is seen that, despite the addition of CUR, a typical peanut-like structure was formed by the porous MnCO_3_ template, as previously found in our studies using the CCD technique [26–29].

There are strict size requirements for particles in drug delivery systems, typically around 100 nm. To enhance cell uptake, it is necessary to reduce the particle size. In this case, the size of MnCO_3_ templates can be reduced by modifying the experimental conditions, such as increasing the salt concentration, decreasing the reaction time and cosurfactant concentration, and changing the solvent type [33]. While nanoparticles are generally more efficiently taken up by cells compared to microparticles, there are instances where the size of drug delivery systems can extend to 1000 nm. This was demonstrated by Bahrom et al., who showed that microparticles with sizes up to 1 µm can be taken up by cells [34]. Thus, submicron particles offer potential in the development of CUR delivery systems.

The photophysical properties of CUR, HSA-MPs, and CUR-HSA-MPs in ethanol solution were evaluated. CUR exhibited the maximum emission at 545 nm, while CUR-HSA-MPs exhibited the maximum emission at 540 nm upon excitation at 488 nm (Figure 2E). This blue shift in emission maximum could be due to the binding of CUR to the hydrophobic core of HSA-MPs, confirming successful entrapment of CUR within the particles. Moreover, HSA-MPs showed no obvious emission maximum, indicating that the fluorescence came from CUR within CUR-HSA-MPs.

**Figure 3:**
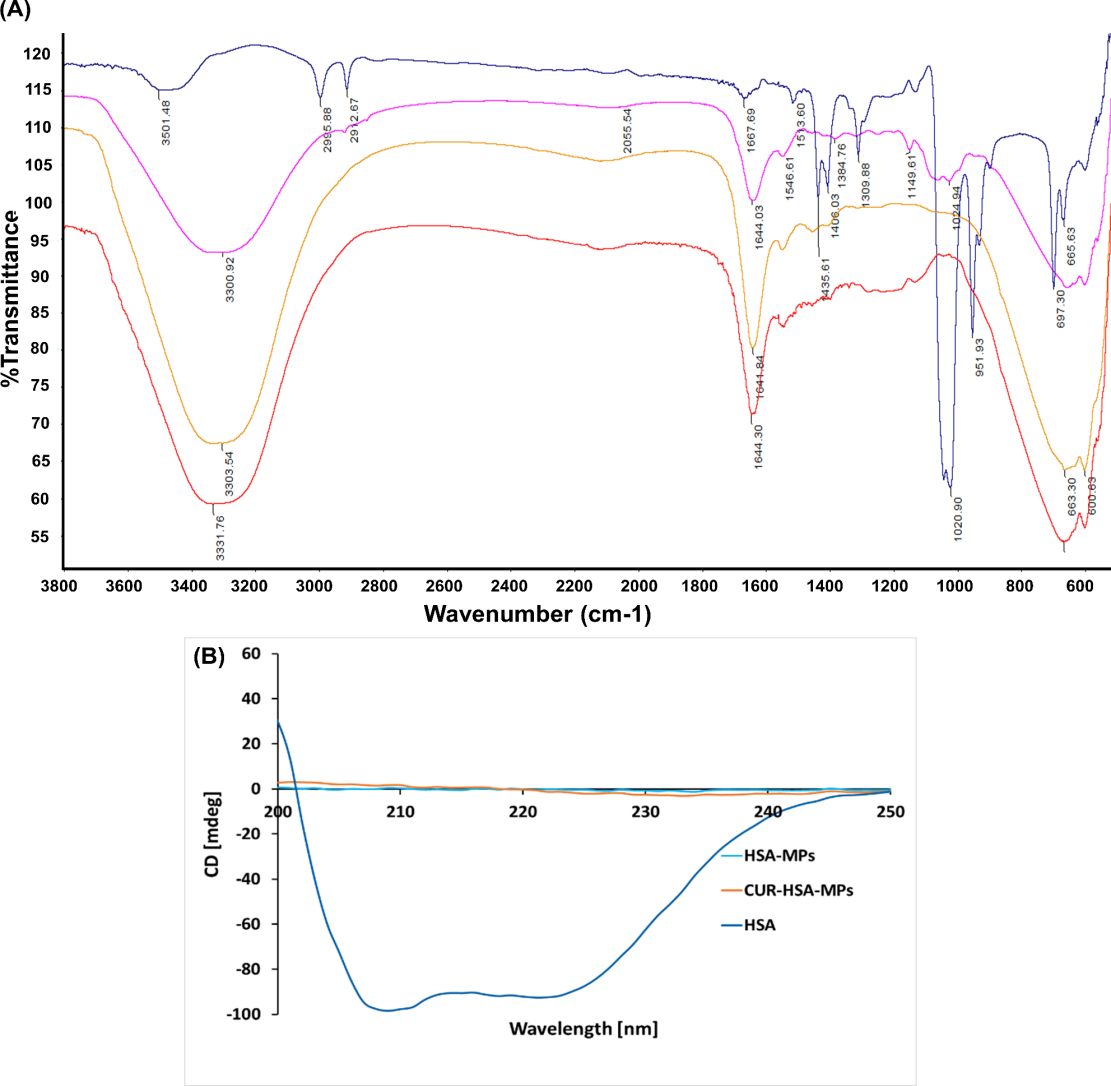
(A) The FTIR spectra of CUR (blue), HSA (pink), HSA-MPs (orange), and CUR-HSA-MPs (red); (B) CD spectra of HSA, HSA-MPs, and CUR-HSA-MPs.

FTIR analyses were used to confirm the chemical binding of CUR to HSA (Figure 3A). The FTIR spectrum of CUR (blue line spectrum) demonstrated bands at 3501 cm^−1^ (broad, phenolic O–H stretching vibration), 1667 cm^−1^ (stretching vibrations of the benzene ring of CUR), 1513 cm^−1^ (C=O and C=C vibrations), and 1435 cm^−1^ (olefinic C–H bending vibration). The 1309 cm^−1^ band corresponds to the aromatic C–O stretching vibrations, while the C–O–C stretching vibrations were represented at 1020/951 cm^−1^ [35]. The characteristic adsorption bands of pure HSA (pink line spectrum) at 1644 and 1546 cm^−1^ indicated the vibration adsorption of amide I (C=O stretching) and amide II (C–N stretching and N–H bending vibrations), respectively. These are the main vibrational bands in the albumin backbone that formed the secondary structure of the protein. It is also seen that the absorption peaks of HSA-MPs (orange line spectrum) have almost the same characteristic peaks of HSA, including amide I and amide II. Interestingly, the similarity between the absorption peaks of HSA-MPs and CUR-HSA-MPs (red line spectrum) suggests that CUR encapsulation does not significantly alter the protein structure of HSA-MPs. Sahoo et al. suggested that CUR analogues do not directly interact with the protein in a way that affects its C–H stretching vibrations or amide I band [36].

Circular dichroism (CD) measurements were carried out to determine changes in the secondary structure of HSA-MPs and CUR-HSA-MPs due to the preparation process, which involves cross-linking of amino acid residues in proteins by glutaraldehyde (GA). As shown in Figure 3B, HSA retained its secondary structure, while both HSA-MPs and CUR-HSA-MPs lost their peptide bonds and secondary structure. The lack of secondary structure of both HSA-MPs and CUR-HSA-MPs is attributed to the cross-linking reaction that utilizes amino acid residues, leading to the destruction of the secondary structure [37].

### Influence of the CUR concentration in the suspension medium on the CUR-loading capacities

**Figure 4:**
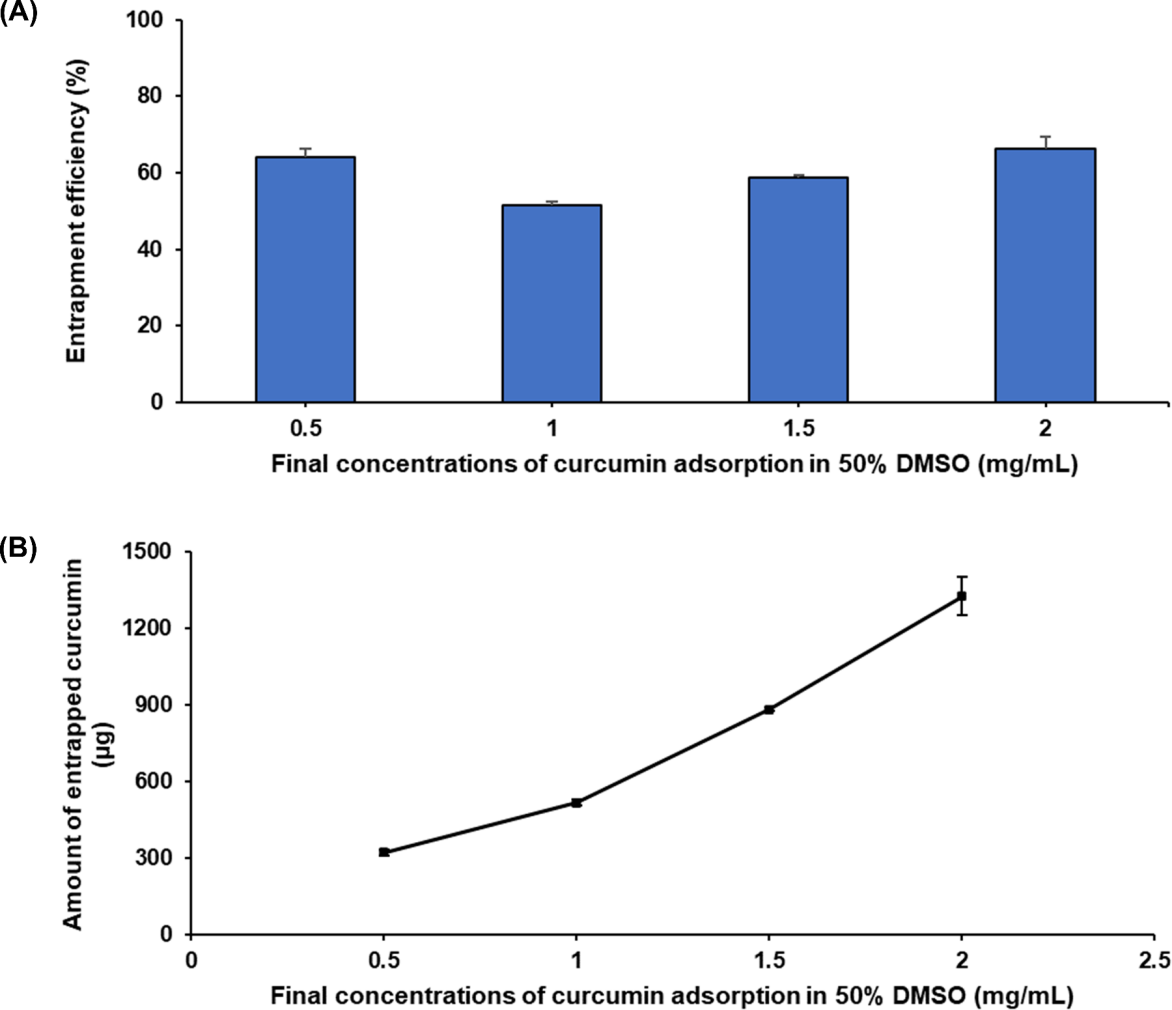
(A) Influence of CUR concentration in the suspension medium on the entrapment efficiency and (B) absolute amount of entrapped CUR in MnCO_3_-HSA-MP solutions. The values are displayed as mean and SD (*n* = 3).

The surface chemical properties of MnCO_3_-HSA-MPs play an important role regarding amount and efficiency of CUR loading. Here, we investigated the influence of the amount of CUR on the %EE. The results are shown in Figure 4A. MnCO_3_-HSA-MPs dispersed in 2 mg/mL of CUR solution displayed the maximum values of EE% and absolute amount of entrapped CUR, whereas those dispersed in 1 mg/mL of CUR solution showed the minimum values of EE%. Those dispersed in 0.5 mg/mL CUR solution demonstrated the lowest values of absolute amount of entrapped CUR.

When increasing the CUR concentration, the EE% started to increase, reached a maximum, and stayed constant. When the CUR concentration reached the maximum of 2 mg/mL, CUR started to precipitate and form aggregates after centrifugation. This led to a miscalculation of the EE% and the absolute amount of entrapped CUR (Figure 4B).

### Release study of CUR from CUR-HSA-MPs

**Figure 5:**
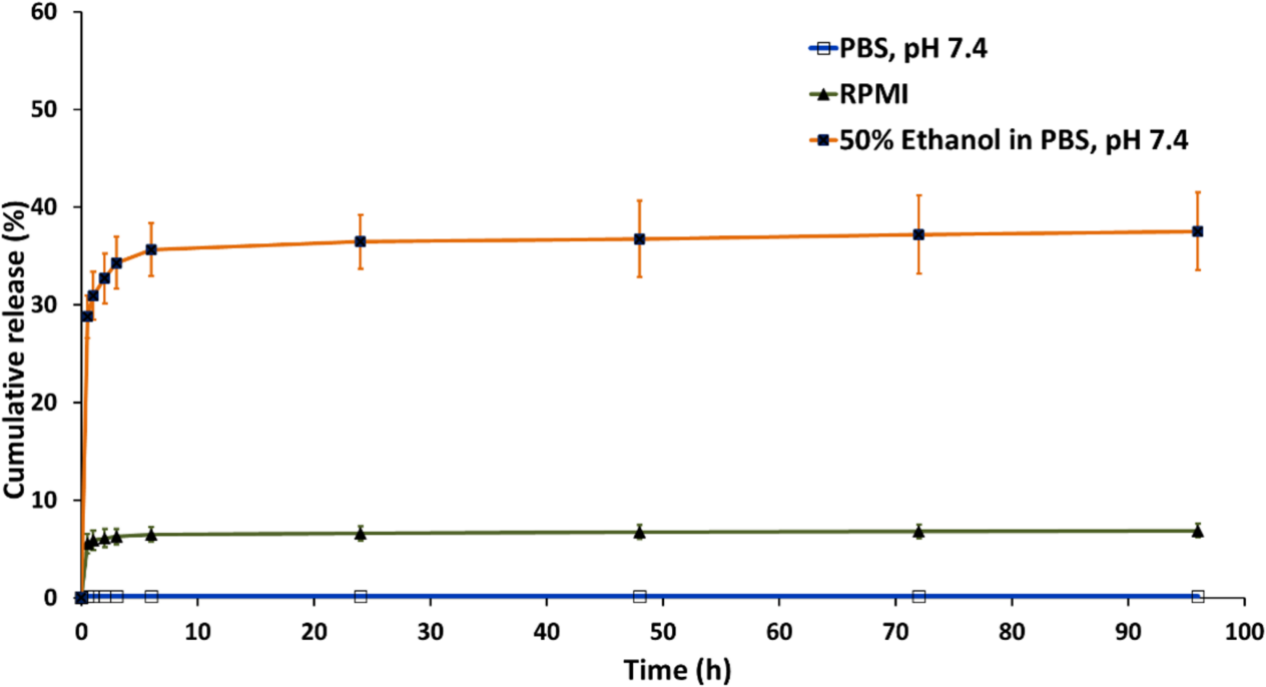
Cumulative release profiles of CUR from CUR-HSA-MPs. The values are displayed as mean and SD (*n* = 3).

CUR-HSA-MPs yielded a sustained release of CUR for 6 h. The cumulative release of CUR was 35% when dispersed in 50% ethanol in PBS at pH 7, and 7% when dispersed in RPMI 1640 medium. However, the release in PBS (pH 7.4) was less than 1% after 96 h, as shown in Figure 5. Sustained release is a desirable property in the treatment of cancer [38–39]. The low percentage of CUR released from CUR-HSA-MPs could be attributed to CUR being covalently bound to the hydrophobic domains of albumin. Another important factor is the shell of GA molecules cross-linking the amino groups on the particles’ surface to form an effective polymeric network, which decreases and prolongs drug release [30].

### In vitro interaction of HSA-MPs and CUR-HSA-MPs with cells

#### Cytotoxicity toward tumor cells

**Figure 6:**
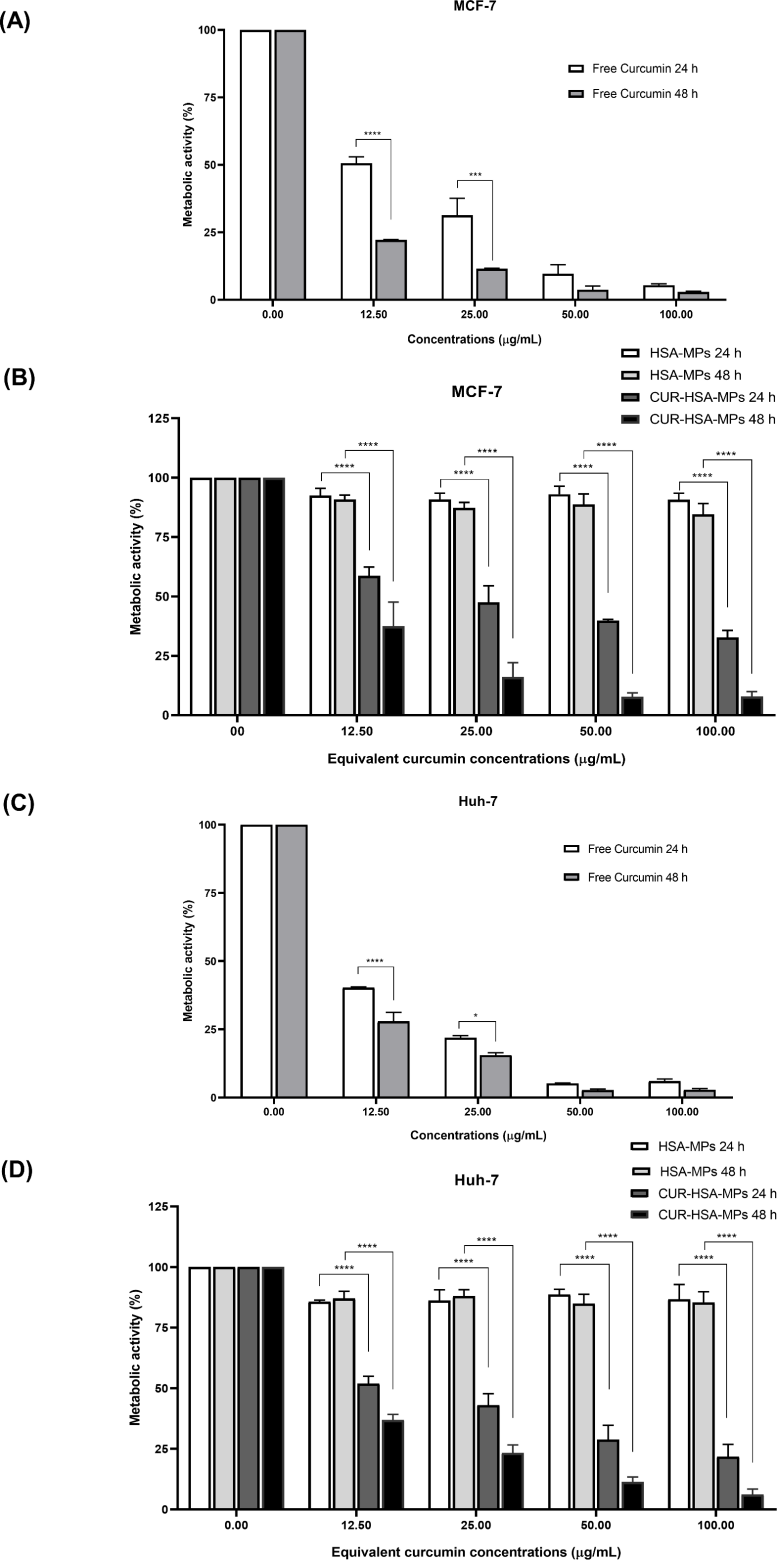
Cytotoxicity of curcumin (A, C), HSA-MPs and CUR-loaded HSA-MPs (B, D), in MFC-7 (A, B) and Huh-7 cells (C, D) compared to the untreated control after 24 and 48 h using the MTT assay. Results are expressed as mean values (*n* = 3) ± SEM. *p < 0.01, ***p < 0.005, ****p < 0.001.

Cytotoxicity of CUR-HSA-MPs in Huh-7 and MCF-7 cancer cell lines was measured using MTT assay. Notably, HSA-MPs revealed no significant cell death among Huh-7 and MCF-7 cells at the concentration range in this experiment (Figure 6B,D). This evidence indicates that the HSA-MPs carrier is non-toxic, even after prolonged exposure.

As shown in Figure 6, free CUR and CUR-HSA-MP treatment substantially induced cell death in a dose- and time-dependent manner. After 24 h, free CUR significantly decreased cell proliferation from 40% to 6% (Huh-7, Figure 6A) and 50% to 5% (MCF-7, Figure 6C) at concentrations of 12.5 and 100 μg/mL, respectively. However, CUR-HSA-MPs, at an equivalent concentration of CUR, significantly decreased cell proliferation from 51% to 21% (Huh-7, Figure 6B) and 58% to 32% (MCF-7, Figure 6D). The CUR-HSA-MPs showed lower cytotoxicity than free CUR at the same drug concentrations. This could be explained by its drug delivery process, which is a common feature of polymer anticancer drugs. Enhanced and sustained release of CUR is attributed to the delayed CUR release from CUR-HSA-MPs enabling long-term drug release, in contrast to the immediate availability of free CUR [40–41]. In the case of slow release rate of MPs, the available CUR concentration is reduced, allowing cells to adapt to stress conditions and thereby showing lower cytotoxicity. However, the cytotoxicity and the interaction of cells with CUR-HSA-MPs depends also on cell uptake of particles and interactions between particles and cells [42].

Our results were in line with those previously reported by Zhang and co-workers [43]. The authors reported that cell viability of A549 cells, HepG2 cells, and RAW264.7 treated with CUR encapsulated in albumin nanoparticles at 100 µg/mL decreased by only 50%, 30%, and 30%, respectively. However, the cell viability after treatment with CUR at the same concentration decreased to less than 7% in all kinds of cells in a 24 h period.

Moreover, after 48 h, both free CUR and CUR-HSA-MPs exhibit stronger toxic effects against Huh-7 than against MCF-7 cells. In line with earlier studies on CUR-loaded gold/chitosan nanogels, a higher concentration-dependent cell viability reduction is induced in Huh-7 cells than in MCF-7 cancerous cells [44]. These findings indicate that the sensitivity to CUR and the CUR-loaded HSA-MPs depends on the cell type [41]. Previous studies have shown that CUR inhibits the growth by inducing apoptosis through p53-dependent Bax induction in MCF-7 breast cancer cells [45–46] or through p38-dependent up-regulation of FasL in Huh7 cells [5].

#### Cytotoxicity toward non-cancer cells

**Figure 7:**
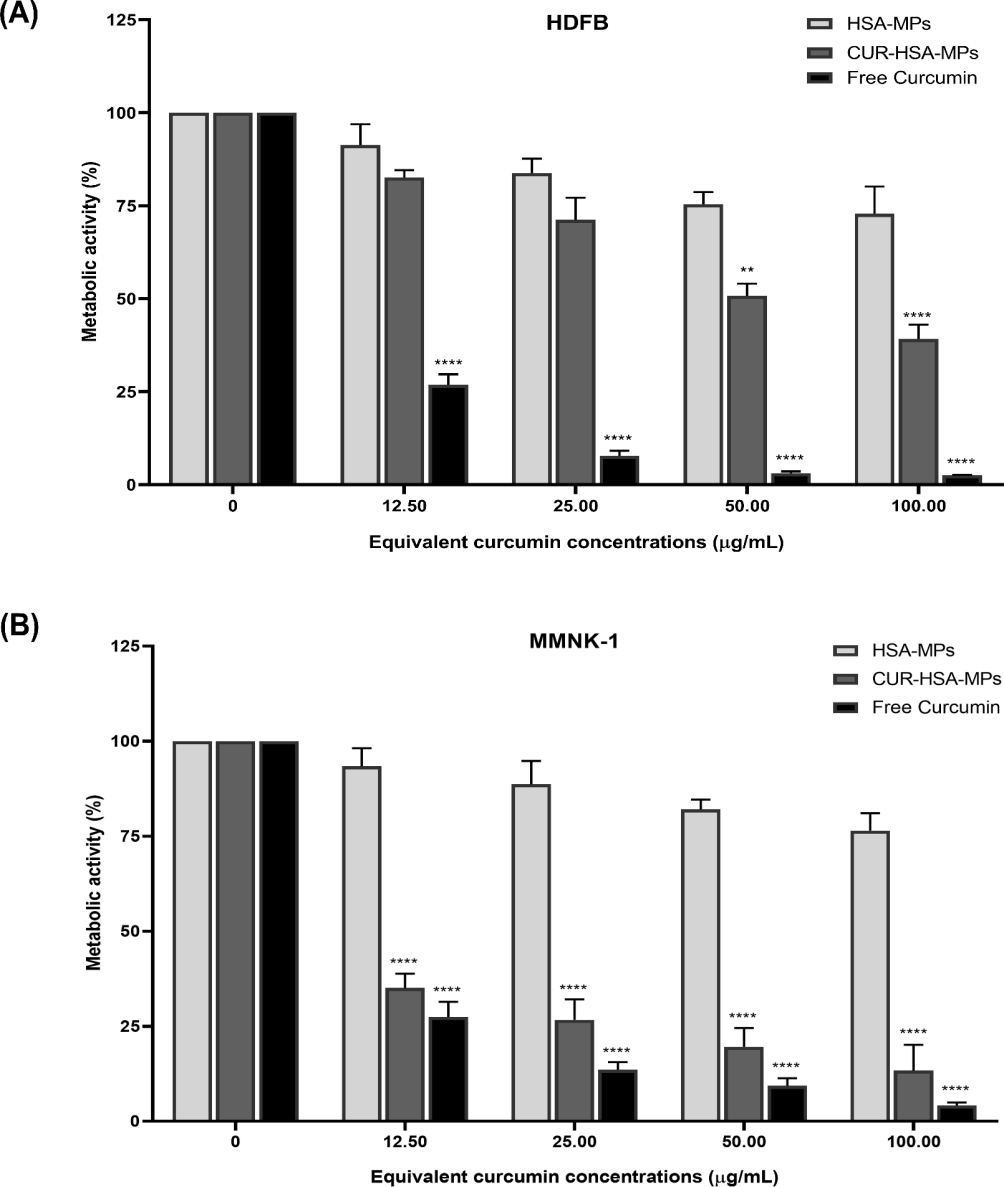
Cytotoxic activity of free curcumin, HSA-MPs, and CUR-HSA-MPs in HDFB (A) and MMNK-1 (B) after 24 h compared to untreated cells (control) using the MTT assay. Results are expressed as mean values (*n* = 3) ± SEM. **p < 0.01, ****p < 0.001.

The cytotoxicity of CUR-HSA-MPs was also evaluated in the non-cancerous cell line HDFB, derived from normal human dermal, fibroblasts and the MMNK-1 human cholangiocyte cell line. The data show that free CUR at lowest concentrations (12.50 μg/mL) had toxic effects toward fibroblasts, whereas CUR-HSA-MPs significantly reduced the toxicity of the free drug (Figure 7A,B). However, when the CUR concentration in CUR-HSA-MPs was increased to 50 μg/mL, a significant loss of viability was observed in fibroblasts. Moreover, CUR-HSA-MPs showed a very strong cytotoxic activity on MMNK-1 cells. Free CUR, however, showed higher cytotoxicity than CUR-HSA-MPs in MMNK-1 cells. In fact, CUR has a cytotoxic effect on normal cells, but tumor cells are more sensitive to it [47].

As demonstrated recently, CUR preferably induces apoptosis in highly proliferating cells. This effect is more pronounced in cancer cells than in healthy ones. As a result, CUR-HSA-MPs have higher specificity and less toxicity against normal cells than cancer cells [48]. Furthermore, the number of albumin receptors on cancer cells is greater than on normal cells, leading to increased albumin endocytosis and a greater uptake of curcumin bound to it into cancer cells [49–50]. Consequently, the use of CUR-HSA-MPs may be a more effective strategy than the use of free CUR for the treatment of cancer.

#### Cell uptake

**Figure 8:**
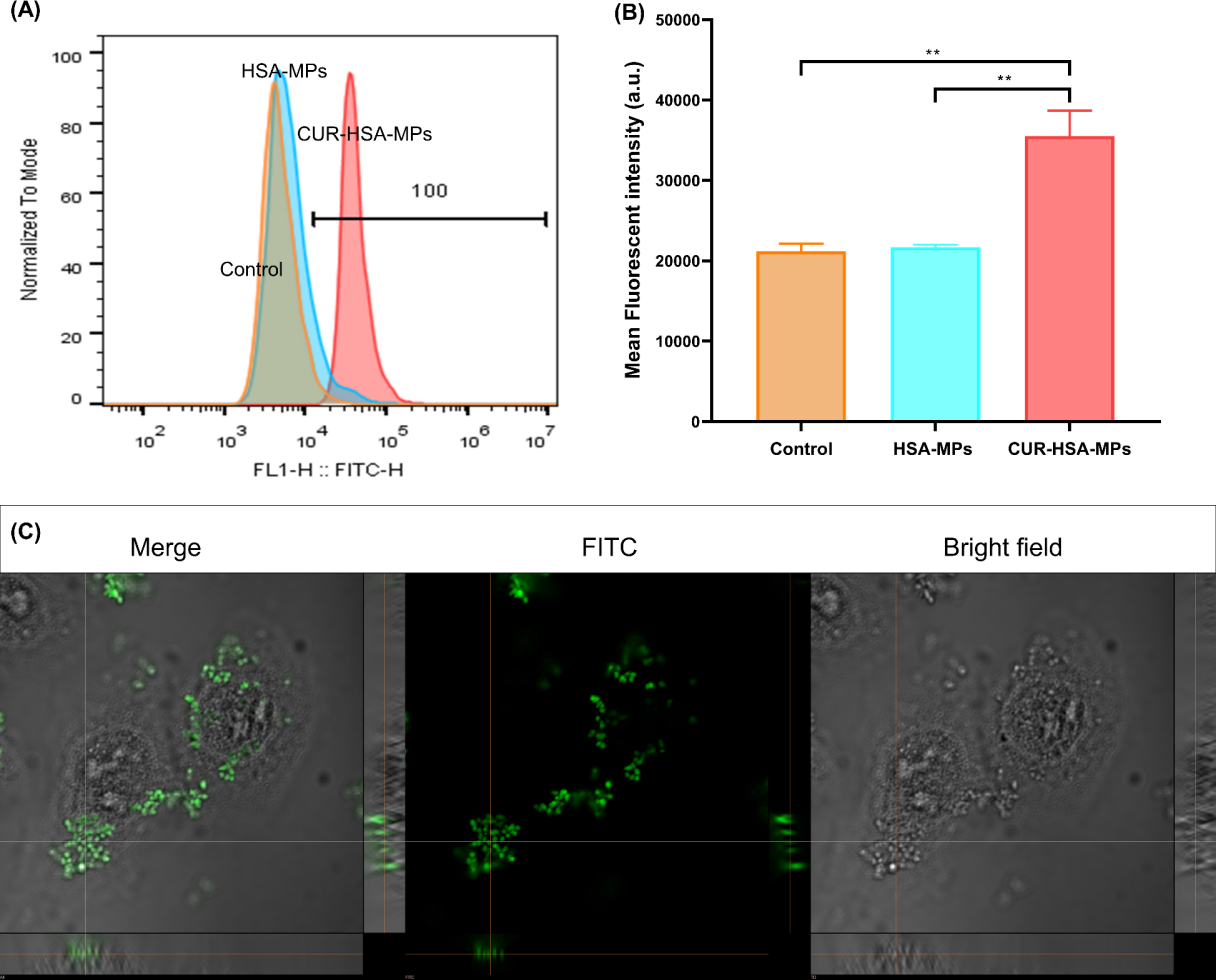
Flow cytometric analysis showing cellular uptake of HSA-MPs and CUR-HSA-MPs at a concentration of 25 µg/mL. (A) Flow cytometry graphs; (B) quantification of the mean fluorescence intensity. Loading curcumin in the albumin submicron particles enhanced its cellular uptake by a factor of 1.6. Results are expressed as mean values (*n* = 3) ± SEM. **p < 0.01; (C) Representative confocal *z*-stack images demonstrating cellular uptake of FITC-CUR-HSA-MPs (an equivalent concentration of curcumin of 25 mg/mL) by MCF-7 cells after 4 h of incubation.

Flow cytometry was employed for the quantitative determination of the cellular uptake of HSA-MPs and CUR-HSA-MPs. The intrinsic fluorescence of both particle types excited at 488 nm allowed for their visualization, as the fluorescence properties were preserved upon encapsulation (Figure 2E). Quantitative analysis of cellular uptake, performed through flow cytometry, revealed a 1.6-fold increase in the uptake of CUR-HSA-MPs compared to HSA-MPs (Figure 8B,C). However, the mean intrinsic fluorescence intensity of HSA-MPs is significantly lower than that of CUR-HSA-MPs when excited at 488 nm, as shown in Figure 2E. Therefore, confirming the preferential uptake of CUR-HSA-MPs by MCF-7 cells is challenging.

Cellular uptake was measured in MCF-7 cells as these cells have a high affinity for albumins. Figure 8C shows a *z*-stack section for FITC-CUR-HSA-MPs, exhibiting green fluorescence due to the uptake of CUR. This demonstrates that CUR-HSA-MPs were readily taken up by cancer cells, likely attributed to particle uptake through endocytosis pathways, including phagocytosis and macropinocytosis [51]. The uptake process depends on cell membrane and particle properties, including size, shape, composition, and surface properties. These factors play a crucial role in particle cellular uptake and internalization [52]. In earlier studies, particles up to 1000 nm were found in various cytoplasmic areas, mainly taken up by cells through endocytic mechanisms [53].

The peanut-shaped microparticles prepared in this study have an aspect ratio (length/width) as high as 1.6. Several studies were carried out to investigate the uptake of non*-*spherical particles. It was demonstrated that the ellipsoidal microparticles with an aspect ratio of approximately 1.6 are internalized to a higher extent by cancer cells compared to toroidal and spheroidal particles with a lower aspect ratio [34]. Similarly, Parakhonskiy et al. showed the same tendency, where ellipsoidal particles with a higher aspect ratio were taken up more effectively by cervical carcinoma cells than more spherical particles with a lower aspect ratio [54]*.* This implies that peanut-shaped particles hold promise for effective uptake into cancer cells, making them a promising option for cancer therapy.

In addition, the surface modification of microparticles using substances such as antibodies and polymers suggests the potential for enhanced uptake, as previously demonstrated [55–56]. Nevertheless, further research should explore to enhance the biological properties and improve cellular uptake of the MPs. The cellular uptake was also measured by fluorescence microscopy as shown in Supporting Information File 1, Figure S1.

## Conclusion

Biodegradable albumin submicron particles were successfully loaded with CUR into protein particles and tested in vitro to evaluate their effects in the treatment of tumors. The amount of CUR entrapped into protein particles was around 55% to 62%. The drug release profile of microparticles demonstrated a release of approximately 37% CUR in a mixture with 50% ethanol, while in RPMI 7% was released, and in PBS, pH 7.4, less than 1% was released after 96 h. The CUR-HSA-MPs exhibited efficacy in inhibiting the cell viability of Huh-7 and MCF-7 cells at a lower-dose treatment; these effects were higher than those in non-cancer cells (HDFB and MMN cells). Moreover, CUR-HSA-MPs could be effectively taken up by MCF-7 cells. The results indicate that the investigated carriers are a highly capable drug delivery system with potential chemotherapeutic applications.

## Experimental

### Materials

CUR (98% purity, from *Curcuma longa*), albumin solution from human serum (100 g/L HSA in 0.14 M NaCl), manganese(II) chloride tetrahydrate (MnCl_2_·4H_2_O), and sodium borohydride (NaBH_4_) were supplied by Sigma-Aldrich Chemical Co. (St. Louis, MO, USA). Sodium carbonate anhydrous (Na_2_CO_3_) and glutaraldehyde (GA, 25% solution in water) were obtained from Merck KGaA (Darmstadt, Germany). Dimethyl sulfoxide (DMSO) was purchased from Panreac Applichem^®^ (Barcelona, Spain). Sodium hydroxide (NaOH), ethylenediaminetetraacetic acid (EDTA) disodium salt dehydrate, and sodium chloride (NaCl) were purchased from HiMedia Laboratories Pvt. Ltd. (Mumbai, India). Thiazolyl blue tetrazolium bromide (MTT powder) and phosphate-buffered saline (PBS), pH 7.4, were purchased from Bio basic (Toronto, Canada). Dulbecco’s modified Eagle’s medium, Ham’s F-12 nutrient mixture (DMEM/F12), and fetal bovine serum (FBS) were purchased from Gibco-BRL Life Technologies (Gibco–Thermo Fisher Scientific, Waltham, MA, USA). Fluorescein isothiocyanate (FITC) was purchased from Biochrom GmbH (Berlin, Germany).

### Fabrication of CUR-loaded human serum albumin microparticles

The CUR-HSA-MPs were prepared by the CCD technique with some modifications to the adsorption method [29]. In brief, 20 mL of 0.25 M MnCl_2_ containing 50 mg/mL HSA solution were mixed with 20 mL of 0.25 M Na_2_CO_3_ under constant vigorous stirring for 30 s at room temperature (RT). HSA solution (0.1%) was subsequently added to the mixture under stirring for 5 min to avoid particle aggregation. The obtained particle suspension was separated by centrifugation (6,000*g* for 3 min). After washing three times with distilled water and adding 5 mL of water, an aliquot of 0.5 mL of particle suspension (a volume concentration of 8%) was then incubated with 2 mg/mL CUR in DMSO (final concentration of CUR and DMSO were 1 mg/mL and 50% (v/v), respectively) for 1 h at RT and protected from light. Then, the mixture was centrifuged and washed three times (6,000*g* for 10 min) with distilled water. The resulting particles were resuspended in 0.9% NaCl and cross-linked with 0.08% GA solution with agitation for 1 h to allow for the cross-linking reaction protected from direct light. After incubation, 0.5 mL of 15 mg/mL of NaBH_4_ in 0.1 M NaOH solution was added to quench the remaining GA in the mixture, and the mixture was incubated for 30 min at RT. Thereafter, EDTA (0.25 M) was added to dissolve the carbonate templates and incubation was performed for 30 min. Finally, the particles were washed three times by centrifugation with distilled water (6,000*g* for 10 min). The obtained HSA particles were resuspended in 10 mL PBS, pH 7.4, and stored in the dark at 4 °C for further use.

The HSA-MPs as control particles were prepared following the same fabrication method but without CUR adsorption.

### Particle characterization

#### Entrapment efficiency of CUR

The entrapment efficiency (EE%) in CUR-HSA-MPs was calculated based on the following equation:


[1] EE%=(CURt−CURf)×100%/CURt

where CURt is the total amount (mass) of CUR in the original incubation solution and CURf is the unloaded CUR amount measured in the supernatant after centrifugation of the loaded CUR. The absorbance was determined with a UV spectrophotometer (Optizen POP, Mecasys Co., Ltd., Daejeon, Korea) at 435 nm. The concentration of CUR was determined using a standard curve of CUR solution.

The fluorescence emission of CUR, HSA-MPs, and CUR-HSA-MPs was evaluated with a Cytation™ 5 Cell Imaging Multi-Mode Reader and analyzed using Gen5 data analysis software (BioTek^®^ Instruments, Inc., Winooski, VT, USA).

#### Particle size and zeta potential

Hydrodynamic size, polydispersity index (PDI), and zeta potential of the particles were evaluated using a Zetasizer Nano ZS (Malvern Instruments Ltd., Malvern, UK) at 25 °C. The suspensions of the MPs were diluted to the appropriate concentration with PBS, pH 7.4 (conductivity 18 mS/cm). The size and zeta potential values were expressed as mean ± standard deviation of at least three repeated measurements.

#### Scanning electron microscopy

The morphology of the particles was examined in a JSM-5910 SEM (JEOL USA, Peabody, MA, USA). The microparticle samples were diluted in distilled water, and a drop of the particle suspension was applied on copper tape and then allowed to dry at RT overnight. The dried samples were then sputter-coated with gold. SEM images were obtained under high-vacuum conditions at an operation voltage of 15 kV.

#### Fourier-transform infrared spectroscopy

Chemical characteristics of CUR, HSA, HSA-MPs, and CUR-HSA-MPs were examined using Fourier-transform infrared spectroscopy (FTIR, Thermo Scientific, NICOLET IS5, Waltham, MA, USA). CUR was prepared in DMSO, while the particles were prepared by dilution in deionized water. The FTIR spectra were recorded in the range of 400–4000 cm^−1^ with a resolution of 4 cm^−1^.

#### Circular dichroism measurements

Circular dichroism (CD) spectra of HSA, HSA-MPs, and CUR- HSA-MPs were recorded using a J-1500 spectropolarimeter (JASCO, Tokyo, Japan) in the wavelength range of 200 to 260 nm. The measurements were conducted at 25 °C in quartz cuvettes with an optical path of 0.1 mm at a scanning speed of 100 nm/min.

### In vitro controlled release studies of CUR-HSA-MPs

A total of 1 mL of the prepared CUR-HSA-MPs (8%) was suspended in 9 mL of either a mixture of 50% ethanol in PBS at pH 7, or PBS at pH 7.4, or RPMI 1640 medium supplemented with 10% fetal bovine serum (FBS) and 1% PenStrep as release medium [57–58]. The solution was continuously stirred at 200 rpm at 37 °C in the dark. Aliquots of 1 mL were taken at appropriate time intervals from 0.5 to 96 h, and the amount taken was replaced with fresh release medium to maintain a constant volume of medium. The aliquots were measured spectrophotometrically at 435 nm to determine the amount of released CUR. The cumulative percent of CUR release for three separate samples was plotted versus time. Ethanol was used in the release medium to enhance solubility and provide sink conditions for poorly water-soluble CUR.

### Interaction of the microparticles with cells

#### Cell culture

The effects of CUR-HSA-MPs on cell cytotoxicity were studied on human hepatocellular carcinoma (Huh-7) and human breast adenocarcinoma (MCF-7) cell lines using the colorimetric MTT assay. Human dermal fibroblasts (HDFB) and human cholangiocyte (MMNK-1) cell lines were evaluated as controls for cytotoxicity.

Briefly, the cell lines were cultured in cell culture medium containing FBS (10%) and penicillin/streptomycin (1%) at 37 °C in a CO_2_ incubator (95% relative humidity, 5% CO_2_). Huh-7, HDFB, and MMNK-1 cells were cultured in DMEM, and MCF-7 cells were cultured in RPMI cell culture medium. The cells were then seeded to grow 24 h prior to treatment at a density of 5 × 10^3^ cells per well in 96-well cell culture plates.

The initial concentrations of free CUR were adjusted to 12.5, 25, 50, or 100 μg/mL in the well plates. The concentration of HSA-MPs and CUR-HSA-MPs suspensions was adjusted to give equivalent concentrations of CUR of 12.5, 25, 50, 100, or 200 μg/mL in the well plates.

Cell cytotoxicity was estimated after 24 and 48 h treatment using the MTT assay. In essence, 10 μL of MTT solution in PBS, pH 7.4 (5 mg/mL), was transferred to each well and incubated for 2 h in a humidified incubator with 5% CO_2_. Mitochondrial enzymes, namely NADPH oxidoreductases, reduce the tetrazolium dye MTT to water-insoluble purple formazan crystals, indicating mitochondrial function and metabolically active cells. After medium removal, the dark blue crystals were dissolved in 100 μL of DMSO; then, the absorption was measured at wavelengths of 570 nm with a Cytation™ 5 Cell Imaging Multi-Mode Reader and analyzed using BioTek Gen5 software.

#### Cellular uptake

To investigate the uptake of particles, HSA-MPs and CUR-HSA-MPs (8%) were labeled with FITC at a ratio of 9:1 at RT for 1 h and protected from light. After incubation, the labeled particles were washed three times with water to remove unbound FITC and resuspended in PBS buffer for further use.

MCF-7 cells were plated at a cell density of 2.5 × 10^4^ cells per well in 8-well plates and allowed to attach for 24 h prior to treatment in a humidified incubator at 37 °C containing 5% CO_2_. Free CUR, HSA-MP, and CUR-HSA-MP suspensions (CUR content equal to 25 and 50 mg/mL) were then added to the cells and incubated for another 4 h. After incubation, the cells were washed with PBS buffer and fixed with freshly prepared 10% paraformaldehyde in PBS followed by two to three washes with PBS, pH 7.4. The uptake was measured under a fluorescent microscope (Nikon ECLIPSE, Ni-U; Nikon, Tokyo, Japan) and confirmed by confocal microscopy (CLSM) (Nikon AX/AX R Confocal Microscope, Tokyo, Japan).

For FACS analysis, cells were trypsinized and washed with PBS three times. Then the samples were examined using a flow cytometer (CytoFLEX, Beckman, USA).

### Statistics

All experiments were performed at least three times. One-way analysis of variance (ANOVA) tests were performed with Tukey post-hoc test in GraphPad Prism software 8 (GraphPad Software, La Jolla, CA, USA). Values were considered statistically significant when *p* was less than 0.05 or 0.01.

## Supporting Information

File 1 Additional experimental data.
