# Supplementary material for: Curcumin-loaded albumin submicron particles with potential as a cancer therapy: an in vitro study
Source: Beilstein J Nanotechnol. 2023 Nov 21;14:1127–40. doi: 10.3762/bjnano.14.93 (PMC10682534; doi:10.3762/bjnano.14.93)
Supplement: File 1 — Additional experimental data. [file Beilstein_J_Nanotechnol-14-1127-s001.pdf]

BEILSTEIN JOURNAL OF NANOTECHNOLOGY

Supporting Information

for

Curcumin-loaded albumin submicron particles with potential as a cancer therapy: an in vitro study

Nittiya Suwannasom, Netsai Sriaksorn, Chutamas Thepmalee, Krissana Khoothiam, Ausanai Prapan, Hans Bäumler and Chonthida Thephinlap

Beilstein J. Nanotechnol. **2023,** *14,* 1127–1140. doi:10.3762/bjnano.14.93

Additional experimental data

License and Terms: This is a supporting information file under the terms of the Creative Commons Attribution License (https://creativecommons.org/licenses/by/4.0). Please note that the reuse, redistribution and reproduction in particular requires that the author(s) and source are credited and that individual graphics may be subject to special legal provisions.
The license is subject to the *Beilstein Journal of Nanotechnology* terms and conditions: (https://www.beilstein-journals.org/bjnano/terms)

Table S1: Size and zeta potential of HSA-MPs and CUR-HSA-MPs.[a]

Particle	Size		Zeta potential		
	Z-Average Size (nm)	PDI	ZP (mV)	Mob (µm·cm/V·s)	Cond (mS/cm)
HSA-MPs	888.88 ± 64	0.3 ± 0.14	−14.50 ± 0.6	−1.12 ± 0.05	18.38 ± 0.18
CUR-HSA-MPs	983.57 ± 82	0.31 ± 0.13	−15.34 ± 0.2	−1.21 ± 0.01	18.10 ± 0.07

[a]Data are presented as mean ± SD (n = 3); conductivity (Cond), electrophoretic mobility unit (Mob), polydispersity index (PDI), and zeta potential (ZP).

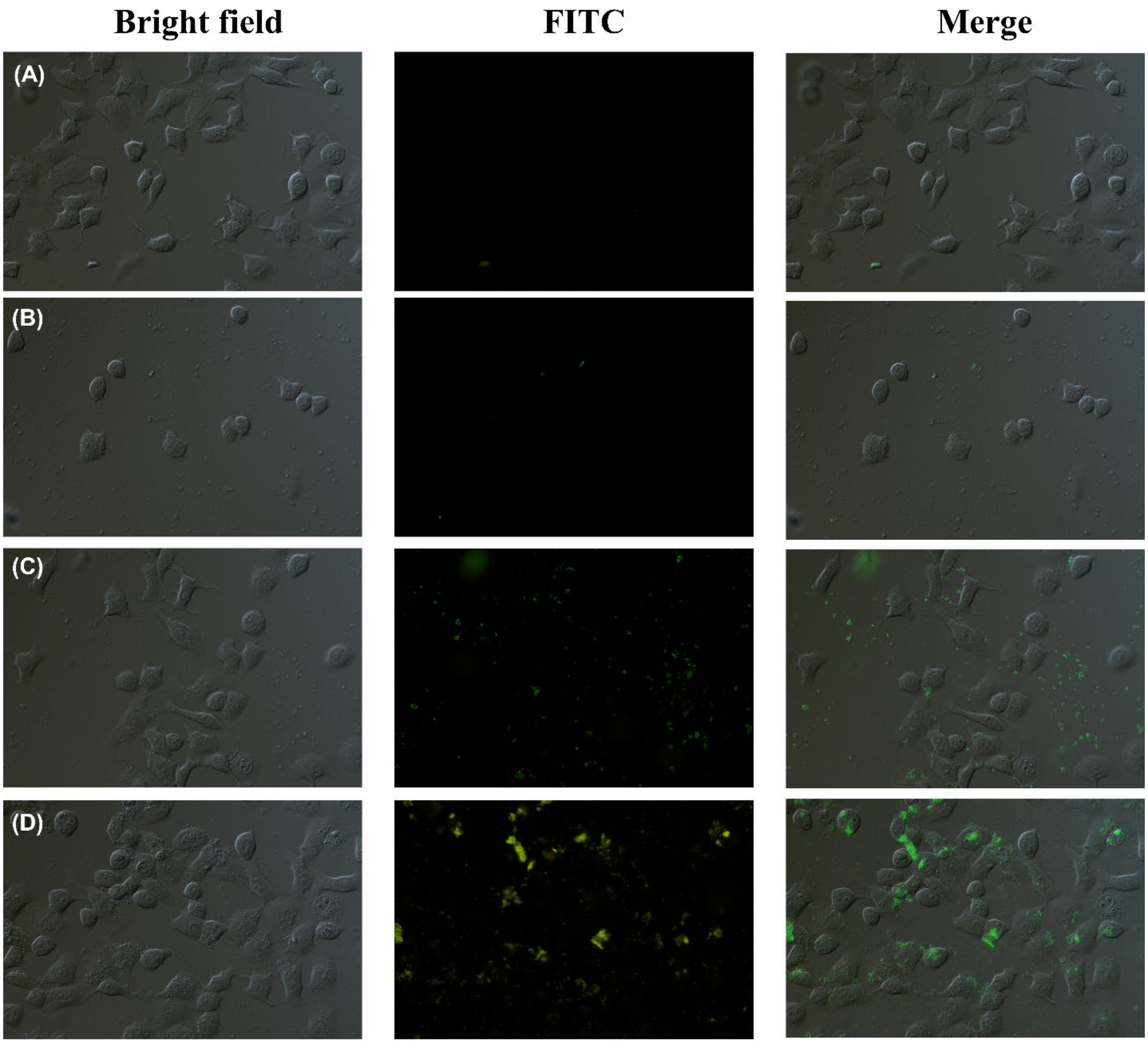

Figure S1: Cellular uptake of CUR at 4 h in MCF-7 cell (A), free CUR (B), FITC-HSA-MPs (C), FITC-CUR-HSA-MPs in MCF-7 cell using fluorescence microscopy. A and B show fluorescent images of non-treated and free curcumin-treated samples to verify the presence of cell autofluorescence. C and D show the uptake of microparticles, which can be seen the fluorescence is diffused throughout the cytoplasm.
